# Pulmonary Hypertension: New Insights and Recent Advances from Basic Science to Translational Approaches

**DOI:** 10.3390/ijms24108462

**Published:** 2023-05-09

**Authors:** Tatyana Novoyatleva

**Affiliations:** Universities of Giessen and Marburg Lung Center (UGMLC), Excellence Cluster Cardio-Pulmonary Institute (CPI), Member of the German Center for Lung Research (DZL), Justus-Liebig University, 35392 Giessen, Germany; tatyana.novoyatleva@innere.med.uni-giessen.de

This Special Issue, “Molecular Research on Pulmonary Hypertension 3.0”, of the *International Journal of Molecular Sciences* (*IJMS*) contains seven original research papers written by experts who highlight recent advances in the field of pulmonary hypertension (PH). PH is a type of high blood pressure that affects the blood vessels of the lungs. Pulmonary arterial hypertension (PAH) is a chronic disease wherein pulmonary arteries become narrowed and blocked, causing vasoconstriction. Upon the disease’s progression, the heart works harder to pump blood through the lungs, which eventually results in right ventricular (RV) failure. PAH is caused by numerous factors, including genetic mutations, autoimmune diseases, congenital heart defects, and certain medications, or it can occur without any known cause, in which case it is referred to as idiopathic PAH. Treatment strategies for PAH vary depending on the severity and underlying cause of the disease, but they are generally based on medications that support the dilation of the pulmonary arteries and improve blood flow. Although appropriate treatment has significantly improved the symptoms, exercise capacity, disease progression, and quality of life related to this disease in recent years, the response to treatment can vary widely among patients, and some of them may not respond to or tolerate certain medications. The research articles contained in this Special Issue summarize and discuss new concepts, mechanistic insights, and strategies for the research and treatment of PAH. Notably, each of these studies can aid the treatment of PAH in the future.

An increasing amount of evidence is indicating the involvement of the immune system in the pathophysiology of PAH. The lungs of most IPAH patients contain elevated levels of various inflammatory cells, which are underlying inflammatory components in PAH patients. Denise van Uden et al. set out to comprehensively characterize the circulating T cells and DCs in patients with IPAH. The authors applied flow cytometry to profile peripheral blood DCs and T cells in treatment-naive IPAH patients and connective tissue disease PAH (CTD-PAH) patients and compared them with healthy control individuals [1]. Through this approach, the authors utilized the unique prospect of identifying possible overlapping pathophysiological features between IPAH and CTD-PAH, the latter being a subtype of PAH associated with CTD that can be caused by autoimmune and inflammatory processes. The authors studied the cytokine-producing capacity, activation marker expression, and subgroup distribution of CD4+ and CD8+ T cells in treatment-naïve IPAH patients at diagnosis and one year after specific PAH treatment. Patients with IPAH showed a decrease in Th2-cell frequency and a significant gain in the expression of the control molecule CTLA4 in naïve CD4+ T cells and in naïve and memory CD8+ T cells. At one-year follow-up, the frequency of IL-17+ production by memory CD4+ T cells was elevated in the patients with IPAH and was accompanied by an increase in the proportion of Th17 and Tc17 cells and a decrease in CTLA4 expression. Principal component analysis was used to distinguish IPAH patients from healthy controls based on T-cell cytokine profiles. Untreated IPAH patients exhibited a unique T-cell phenotype that differed from CTD-PAH patients and was characterized by reduced cytokine-producing capacity. These findings indicate the involvement of adaptive immune responses in IPAH, which may have implications for therapeutic intervention design [1]. In their original article, Leandro C. and co-authors used sheep as a model for transcatheter Percutaneous Pulmonary Artery Laser Denervation (PADN) and found that an ovine model with certain technical and instrumentational precautions may be used for PADN [2]. The authors performed the analysis of PA nerve distribution and noted that ovine nerve distribution in the pulmonary artery bifurcation and main branches differentiate from the innervation of humans with respect to the nerve density in the pulmonary artery trunk, bifurcation, and main branches, which are much lower than in human pulmonary arteries. Specifically, the nerve fibers in sheep were detected in the media layer, adventitia, and perivascular tissue. Therefore, ovine PADN models should be used with caution since the required lesion depth for sheep pulmonary arteries should be less than that in human pulmonary arteries. The authors established that the emergence of lesions is one of the most effective signs of efficient PADN [2].

Given the presence of variable forms of PH and the many existing treatment options, the search for new biomarkers that can accurately determine phenotypes and predict the prognosis of patients in order to guide treatment strategies is a pressing demand. In the manuscript by Diekmann et al., the plasma levels of the soluble receptor for advanced glycation products (sRAGE) were compared in two patient cohorts: adult and pediatric. sRAGE is a soluble form of the cell surface receptor for advanced glycation end products (RAGE) that is involved in various cellular processes, including hypoxia, inflammation, oxidative stress, and cell proliferation. In adults, it was found that B-type natriuretic peptide (NTproBNP), Interleukin-6 (IL-6), and sRAGE plasma levels are elevated in adult IPAH and CTD-PAH patients compared to healthy control subjects. All three biomarkers were elevated in WHO functional class II+III PAH patients compared to controls, while sRAGE demonstrated diagnostic accuracy comparable and even superior to that of prognostic NTproBNP in the distinction of PH functional class I. In the pediatric cohort of patients, no difference between plasma sRAGE levels between the PH patients and the non-PH controls was found. Overall, the authors maintained that plasma RAGE serves as a sensitive and accurate PAH biomarker offering superior indices than NTproBNP for the distinction of mild PAH cases from controls [3].

Hereditary PAH (HPAH), which is inherited in an autosomal-dominant manner, is a rare genetic disorder caused by mutations in various genes, including potassium channel subfamily K member 3 (KCNK3). The study by West et al. aimed to investigate various stressors driving the susceptibility of patients with a KCNK3 mutation to PAH [4]. Although mice with KCNK3 knocked out as adults lacked increased susceptibility to hypoxia and western diet defined by the absence of changes in right ventricular systolic pressure (RVSP). The RNASeq analyses of these lungs as well as human induced pluripotent stem cell (iPS) lines of patients with a KCNK3 mutation show changes related to tone, metabolism, and inflammation. Upon low-dose administration, the surviving *kcnk3^fl/fl^* mice showed elevated RVSP and strongly increased muscularization of small vessels, which were linked to heightened susceptibility to inflammatory insult, which is defined by an increase in the levels of pulmonary inflammatory cells and multiple cytokines. Similarly, PAH patients with KCNK3 mutation presented increased cytokines and chronic immune cell activation. Taken together, the authors concluded that KCNK3 mutation results in a predisposition for increased inflammation along multiple axes [4]. Changes in microRNA (miR) expression levels contribute to the emergence and development of PH. Specifically, miR-124 contributes to the pro-proliferative and pro-inflammatory phenotypes of pulmonary vascular cells, while the reduced expression of miRNAs can be successfully re-induced by the specific inhibitors of histone deacetylases (HDACis). Zhang et al. found that HDACi could restore diminished levels of several mature miRNAs, including miR-124, let-7i, miR-224, and miR-210, in pulmonary artery fibroblasts isolated from IPAH patients. Furthermore, HDACi could restore the levels of miR-124 precursors, which were similarly diminished in the pulmonary artery fibroblasts of PH patients. Mechanistically, the authors recognized that HDACi relaxed the condensed miR-124-1 chromatin structure in human PH pulmonary fibroblasts, indicating that epigenetic mechanisms play an essential role in controlling miR-124 in disease and that HDACs might serve as promising therapeutic targets for treating PAH [5].

In this Special Issue, Habbout et al. sought to investigate the role of Enhancer of Zeste Homolog 2 (EZH2), which is a catalytic subunit of the polycomb repressive complex 2 (PRC2) that mediates H3K27 methylation and gene silencing and recently attracted the attention of researchers as a new target for cancer treatment. The authors found that EZH2 was overexpressed in experimental and clinical PAH, specifically in the isolated pulmonary arterial smooth muscle cells (PASMCs) of PAH patients [6]. The blockage of EZH2 by chemical agents or ablation through siRNA-mediated approach obstructed the pro-proliferative and apoptosis-resistant phenotype of PAH pulmonary arterial smooth muscle cells (PASMCs). In contrast, EZH2’s overexpression contributes to the hyper-proliferative and apoptosis-resistant state of PAH-PASMCs affecting both canonical and non-canonical mechanisms. High-throughput RNA sequencing indicated that EZH2 contributes to cell cycle progression by affecting the transcriptome of the E2F pathway and via the direct involvement of E2F1 in the EZH2-mediated upregulation of gene expression. Quantitative proteomic analysis of PASMCs suggested that EZH2 is essential for maintaining bioenergetic machinery and oxidative phosphorylation, as amongst the detected proteins affected by EZH2 ablation were those that were enriched in pathways involved in the tricarboxylic acid (TCA) cycle and mitochondrial translation machinery [6]. 

Despite many therapeutic advances, PAH remains an incurable disease with high morbidity and early mortality. As the discovery of de novo drugs is a costly and time-consuming process, the repurposing of existing medications constitutes a desirable strategy for the treatment of PAH. Trifluoperazine (TFP), an antipsychotic drug, has been reported to exert anti-proliferative and anti-survival effects in various cancer models. Grobs and co-authors investigated the impact of TFP on PAH. Importantly, it has been revealed that TFP has both anti-survival and anti-proliferative effects on cultured PAH-PASMCs, which were associated with the induction of autophagy. The authors suggested that TFP-induced autophagy represents an unsuccessful protective mechanism. The administration of TFP in two experimental rat models (Monocrotaline- and Sugen/hypoxia-induced PAH) significantly lowered RVSP, diminished total pulmonary resistance, decreased the medial wall thickness of distal pulmonary arteries, and improved RV function. Overall, these data indicate that TFP might provide beneficial effects for the treatment of PAH and support the notion that finding new applications for old medications could be a very productive approach [7].
